# Prostate cancer risk stratification improvement across multiple ancestries with new polygenic hazard score

**DOI:** 10.1038/s41391-022-00497-7

**Published:** 2022-02-12

**Authors:** Minh-Phuong Huynh-Le, Roshan Karunamuni, Chun Chieh Fan, Lui Asona, Wesley K. Thompson, Maria Elena Martinez, Rosalind A. Eeles, Zsofia Kote-Jarai, Kenneth R. Muir, Artitaya Lophatananon, Johanna Schleutker, Nora Pashayan, Jyotsna Batra, Henrik Grönberg, David E. Neal, Børge G. Nordestgaard, Catherine M. Tangen, Robert J. MacInnis, Alicja Wolk, Demetrius Albanes, Christopher A. Haiman, Ruth C. Travis, William J. Blot, Janet L. Stanford, Lorelei A. Mucci, Catharine M. L. West, Sune F. Nielsen, Adam S. Kibel, Olivier Cussenot, Sonja I. Berndt, Stella Koutros, Karina Dalsgaard Sørensen, Cezary Cybulski, Eli Marie Grindedal, Florence Menegaux, Jong Y. Park, Sue A. Ingles, Christiane Maier, Robert J. Hamilton, Barry S. Rosenstein, Yong-Jie Lu, Stephen Watya, Ana Vega, Manolis Kogevinas, Fredrik Wiklund, Kathryn L. Penney, Chad D. Huff, Manuel R. Teixeira, Luc Multigner, Robin J. Leach, Hermann Brenner, Esther M. John, Radka Kaneva, Christopher J. Logothetis, Susan L. Neuhausen, Kim De Ruyck, Piet Ost, Azad Razack, Lisa F. Newcomb, Jay H. Fowke, Marija Gamulin, Aswin Abraham, Frank Claessens, Jose Esteban Castelao, Paul A. Townsend, Dana C. Crawford, Gyorgy Petrovics, Ron H. N. van Schaik, Marie-Élise Parent, Jennifer J. Hu, Wei Zheng, UKGPCS collaborators, APCB (Australian Prostate Cancer BioResource), NC-LA PCaP Investigators, The IMPACT Study Steering Committee and Collaborators, Canary PASS Investigators, The Profile Study Steering Committee, The PRACTICAL Consortium, Ian G. Mills, Ole A. Andreassen, Anders M. Dale, Tyler M. Seibert

**Affiliations:** 1 Radiation Oncology, George Washington University, Washington, DC USA; 2 Department of Radiation Medicine and Applied Sciences, University of California San Diego, La Jolla, CA USA; 3 Center for Multimodal Imaging and Genetics, University of California San Diego, La Jolla, CA USA; 4 Division of Biostatistics and Halicioğlu Data Science Institute, University of California San Diego, La Jolla, CA USA; 5 Department of Family Medicine and Public Health, University of California San Diego, La Jolla, CA USA; 6 University of California San Diego, Moores Cancer Center, Herbert Wertheim School of Public Health and Human Longevity Science, University of California San Diego, La Jolla, CA 92093-0012 USA; 7 The Institute of Cancer Research, London, SM2 5NG UK; 8 Royal Marsden NHS Foundation Trust, London, SW3 6JJ UK; 9 Division of Population Health, Health Services Research and Primary Care, University of Manchester, Oxford Road, Manchester, M13 9PL UK; 10 Institute of Biomedicine, University of Turku, Turku, Finland; 11 Department of Medical Genetics, Genomics, Laboratory Division, Turku University Hospital, PO Box 52, 20521 Turku, Finland; 12 Department of Applied Health Research, University College London, London, WC1E 7HB UK; 13 Centre for Cancer Genetic Epidemiology, Department of Oncology, University of Cambridge, Strangeways Laboratory, Worts Causeway, Cambridge, CB1 8RN UK; 14 Australian Prostate Cancer Research Centre-Qld, Institute of Health and Biomedical Innovation and School of Biomedical Sciences, Queensland University of Technology, Brisbane, QLD 4059 Australia; 15 Translational Research Institute, Brisbane, QLD 4102 Australia; 16 Department of Medical Epidemiology and Biostatistics, Karolinska Institute, SE-171 77 Stockholm, Sweden; 17 Nuffield Department of Surgical Sciences, University of Oxford, Room 6603, Level 6, John Radcliffe Hospital, Headley Way, Headington, Oxford, OX3 9DU UK; 18 University of Cambridge, Department of Oncology, Box 279, Addenbrooke’s Hospital, Hills Road, Cambridge, CB2 0QQ UK; 19 Cancer Research UK, Cambridge Research Institute, Li Ka Shing Centre, Cambridge, CB2 0RE UK; 20 Faculty of Health and Medical Sciences, University of Copenhagen, 2200 Copenhagen, Denmark; 21 Department of Clinical Biochemistry, Herlev and Gentofte Hospital, Copenhagen University Hospital, Herlev, 2200 Copenhagen Denmark; 22 SWOG Statistical Center, Fred Hutchinson Cancer Research Center, Seattle, WA USA; 23 Cancer Epidemiology Division, Cancer Council Victoria, 615 St Kilda Road, Melbourne, VIC 3004 Australia; 24 Centre for Epidemiology and Biostatistics, Melbourne School of Population and Global Health, The University of Melbourne, Grattan Street, Parkville, VIC 3010 Australia; 25 Department of Surgical Sciences, Uppsala University, 75185 Uppsala, Sweden; 26 Division of Cancer Epidemiology and Genetics, National Cancer Institute, NIH, Bethesda, MD 20892 USA; 27 Center for Genetic Epidemiology, Department of Preventive Medicine, Keck School of Medicine, University of Southern California/Norris Comprehensive Cancer Center, Los Angeles, CA 90015 USA; 28 Cancer Epidemiology Unit, Nuffield Department of Population Health, University of Oxford, Oxford, OX3 7LF UK; 29 Division of Epidemiology, Department of Medicine, Vanderbilt University Medical Center, 2525 West End Avenue, Suite 800, Nashville, TN 37232 USA; 30 International Epidemiology Institute, Rockville, MD 20850 USA; 31 Division of Public Health Sciences, Fred Hutchinson Cancer Research Center, Seattle, WA 98109-1024 USA; 32 Department of Epidemiology, School of Public Health, University of Washington, Seattle, WA 98195 USA; 33 Department of Epidemiology, Harvard T. H. Chan School of Public Health, Boston, MA 02115 USA; 34 Division of Cancer Sciences, University of Manchester, Manchester Academic Health Science Centre, Radiotherapy Related Research, The Christie Hospital NHS Foundation Trust, Manchester, M13 9PL UK; 35 Division of Urologic Surgery, Brigham and Womens Hospital, 75 Francis Street, Boston, MA 02115 USA; 36 Sorbonne Universite, GRC n°5, AP-HP, Tenon Hospital, 4 rue de la Chine, F-45020 Paris, France; 37 CeRePP, Tenon Hospital, F-75020 Paris, France; 38 Department of Molecular Medicine, Aarhus University Hospital, Palle Juul-Jensen Boulevard 99, 8200 Aarhus N, Denmark; 39 Department of Clinical Medicine, Aarhus University, DK 8200 Aarhus N, Denmark; 40 International Hereditary Cancer Center, Department of Genetics and Pathology, Pomeranian Medical University, 70-115 Szczecin, Poland; 41 Department of Medical Genetics, Oslo University Hospital, 0424 Oslo, Norway; 42 Exposome and Heredity, CESP (UMR 1018), Faculté de Médecine, Université Paris-Saclay, Inserm, Gustave Roussy, Villejuif, France; 43 Department of Cancer Epidemiology, Moffitt Cancer Center, 12902 Magnolia Drive, Tampa, FL 33612 USA; 44 Department of Preventive Medicine, Keck School of Medicine, University of Southern California/Norris Comprehensive Cancer Center, Los Angeles, CA 90015 USA; 45 Humangenetik Tuebingen, Paul-Ehrlich-Str 23, D-72076 Tuebingen, Germany; 46 Dept. of Surgical Oncology, Princess Margaret Cancer Centre, Toronto, ON M5G 2M9 Canada; 47 Dept. of Surgery (Urology), University of Toronto, Toronto, Canada; 48 Department of Radiation Oncology and Department of Genetics and Genomic Sciences, Box 1236, Icahn School of Medicine at Mount Sinai, One Gustave L. Levy Place, New York, NY 10029 USA; 49 Centre for Cancer Biomarker and Biotherapeutics, Barts Cancer Institute, Queen Mary University of London, John Vane Science Centre, Charterhouse Square, London, EC1M 6BQ UK; 50 Uro Care, Kampala, Uganda; 51 Fundación Pública Galega Medicina Xenómica, Santiago de Compostela, 15706 Spain; 52 Instituto de Investigación Sanitaria de Santiago de Compostela, Santiago De Compostela, 15706 Spain; 53 Centro de Investigación en Red de Enfermedades Raras (CIBERER), Santiago De Compostela, Spain; 54 ISGlobal, Barcelona, Spain; 55 IMIM (Hospital del Mar Medical Research Institute), Barcelona, Spain; 56 Universitat Pompeu Fabra (UPF), Barcelona, Spain; 57 Channing Division of Network Medicine, Department of Medicine, Brigham and Women’s Hospital/Harvard Medical School, Boston, MA 02115 USA; 58 Department of Epidemiology, The University of Texas MD Anderson Cancer Center, 1515 Holcombe Blvd., Houston, TX 77030 USA; 59 Department of Genetics, Portuguese Oncology Institute of Porto (IPO-Porto), 4200-072 Porto, Portugal; 60 Biomedical Sciences Institute (ICBAS), University of Porto, 4050-313 Porto, Portugal; 61 Cancer Genetics Group, IPO-Porto Research Center (CI-IPOP), Portuguese Oncology Institute of Porto (IPO-Porto), 4200-072 Porto, Portugal; 62 Univ Rennes, Inserm, EHESP, Irset (Institut de recherche en santé, environnement et travail) - UMR_S 1085, Rennes, France; 63 Department of Cell Systems and Anatomy, Mays Cancer Center, University of Texas Health Science Center at San Antonio, San Antonio, TX USA; 64 Division of Clinical Epidemiology and Aging Research, German Cancer Research Center (DKFZ), D-69120 Heidelberg, Germany; 65 German Cancer Consortium (DKTK), German Cancer Research Center (DKFZ), D-69120 Heidelberg, Germany; 66 Division of Preventive Oncology, German Cancer Research Center (DKFZ) and National Center for Tumor Diseases (NCT), Im Neuenheimer Feld 460, 69120 Heidelberg, Germany; 67 Departments of Epidemiology & Population Health and of Medicine, Division of Oncology, Stanford Cancer Institute, Stanford University School of Medicine, Stanford, CA 94304 USA; 68 Molecular Medicine Center, Department of Medical Chemistry and Biochemistry, Medical University of Sofia, Sofia, 2 Zdrave Str., 1431 Sofia, Bulgaria; 69 The University of Texas M. D. Anderson Cancer Center, Department of Genitourinary Medical Oncology, 1515 Holcombe Blvd., Houston, TX 77030 USA; 70 Department of Population Sciences, Beckman Research Institute of the City of Hope, 1500 East Duarte Road, Duarte, CA 91010 USA; 71 Ghent University, Faculty of Medicine and Health Sciences, Basic Medical Sciences, Proeftuinstraat 86, B-9000 Gent, Belgium; 72 Ghent University Hospital, Department of Radiotherapy, De Pintelaan 185, B-9000 Gent, Belgium; 73 Department of Surgery, Faculty of Medicine, University of Malaya, 50603 Kuala Lumpur, Malaysia; 74 Department of Urology, University of Washington, 1959 NE Pacific Street, Box 356510, Seattle, WA 98195 USA; 75 Division of Epidemiology, Department of Preventive Medicine, University of Tennessee Health Science Center, Memphis, TN 38163 USA; 76 Department of Oncology, University Hospital Centre Zagreb, University of Zagreb, School of Medicine, 10 000 Zagreb, Croatia; 77 Department of Oncology, Cross Cancer Institute, University of Alberta, 11560 University Avenue, Edmonton, AB T6G 1Z2 Canada; 78 Molecular Endocrinology Laboratory, Department of Cellular and Molecular Medicine, KU Leuven, BE-3000 Belgium; 79 Genetic Oncology Unit, CHUVI Hospital, Complexo Hospitalario Universitario de Vigo, Instituto de Investigación Biomédica Galicia Sur (IISGS), 36204 Vigo (Pontevedra), Spain; 80 Division of Cancer Sciences, Manchester Cancer Research Centre, Faculty of Biology, Medicine and Health, Manchester Academic Health Science Centre, NIHR Manchester Biomedical Research Centre, Health Innovation Manchester, Univeristy of Manchester, Manchester, M13 9WL UK; 81 The University of Surrey, Guildford, Surrey GU2 7XH UK; 82 Case Western Reserve University, Department of Population and Quantitative Health Sciences, Cleveland Institute for Computational Biology, 2103 Cornell Road, Wolstein Research Building, Suite 2527, Cleveland, OH 44106 USA; 83 Uniformed Services University, 4301 Jones Bridge Rd, Bethesda, MD 20814 USA; 84 Center for Prostate Disease Research, 6720A Rockledge Drive, Suite 300, Bethesda, MD 20817 USA; 85 Department of Clinical Chemistry, Erasmus University Medical Center, 3015 CE Rotterdam, The Netherlands; 86 Epidemiology and Biostatistics Unit, Centre Armand-Frappier Santé Biotechnologie, Institut national de la recherche scientifique, 531 Boul. des Prairies, Laval, QC H7V 1B7 Canada; 87 Department of Social and Preventive Medicine, School of Public Health, University of Montreal, Montreal, QC Canada; 88 The University of Miami School of Medicine, Sylvester Comprehensive Cancer Center, 1120 NW 14th Street, CRB 1511, Miami, FL 33136 USA; 89 Nuffield Department of Surgical Sciences, University of Oxford, Oxford, UK; 90 NORMENT, KG Jebsen Centre, Oslo University Hospital and University of Oslo, Oslo, Norway; 91 Department of Radiology, University of California San Diego, La Jolla, CA USA; 92 Department of Bioengineering, University of California San Diego, La Jolla, CA USA; 93 http://www.icr.ac.uk/our-research/research-divisions/division-of-genetics-and-epidemiology/oncogenetics/research-projects/ukgpcs/ukgpcs-collaborators; 94 Australian Prostate Cancer Research Centre-Qld, Queensland University of Technology, Brisbane; Prostate Cancer Research Program, Monash University, Melbourne; Dame Roma Mitchell Cancer Centre, University of Adelaide, Adelaide; Chris O’Brien Lifehouse and The Kinghorn Cancer Centre, Sydney, NSW Australia; 95 Translational Research Institute, Brisbane, QLD Australia; 96 https://pcap.bioinf.unc.edu/; 97 http://impact.icr.ac.uk; 98 http://www.cancerresearchuk.org/about-cancer/find-a-clinical-trial/a-study-find-out-looking-gene-changes-would-be-useful-in-screening-for-prostate-cancer-profile-pilot

**Keywords:** Cancer epidemiology, Cancer screening, Cancer genetics

## Abstract

**Background:**

Prostate cancer risk stratification using single-nucleotide polymorphisms (SNPs) demonstrates considerable promise in men of European, Asian, and African genetic ancestries, but there is still need for increased accuracy. We evaluated whether including additional SNPs in a prostate cancer polygenic hazard score (PHS) would improve associations with clinically significant prostate cancer in multi-ancestry datasets.

**Methods:**

In total, 299 SNPs previously associated with prostate cancer were evaluated for inclusion in a new PHS, using a LASSO-regularized Cox proportional hazards model in a training dataset of 72,181 men from the PRACTICAL Consortium. The PHS model was evaluated in four testing datasets: African ancestry, Asian ancestry, and two of European Ancestry—the Cohort of Swedish Men (COSM) and the ProtecT study. Hazard ratios (HRs) were estimated to compare men with high versus low PHS for association with clinically significant, with any, and with fatal prostate cancer. The impact of genetic risk stratification on the positive predictive value (PPV) of PSA testing for clinically significant prostate cancer was also measured.

**Results:**

The final model (PHS290) had 290 SNPs with non-zero coefficients. Comparing, for example, the highest and lowest quintiles of PHS290, the hazard ratios (HRs) for clinically significant prostate cancer were 13.73 [95% CI: 12.43–15.16] in ProtecT, 7.07 [6.58–7.60] in African ancestry, 10.31 [9.58–11.11] in Asian ancestry, and 11.18 [10.34–12.09] in COSM. Similar results were seen for association with any and fatal prostate cancer. Without PHS stratification, the PPV of PSA testing for clinically significant prostate cancer in ProtecT was 0.12 (0.11–0.14). For the top 20% and top 5% of PHS290, the PPV of PSA testing was 0.19 (0.15–0.22) and 0.26 (0.19–0.33), respectively.

**Conclusions:**

We demonstrate better genetic risk stratification for clinically significant prostate cancer than prior versions of PHS in multi-ancestry datasets. This is promising for implementing precision-medicine approaches to prostate cancer screening decisions in diverse populations.

## Introduction

Identification of men at greatest risk of developing prostate cancer remains an important challenge. Risk stratification using common genetic markers, such as single-nucleotide polymorphisms (SNPs), shows promise toward more effectively identifying men at greatest risk of developing aggressive or fatal prostate cancer [1, 2]. Analyses of benefit, harm, and cost-effectiveness support use of genomic risk stratification to guide prostate cancer screening [3, 4]. Ensuring these tools perform well in diverse populations is important to ensure risk stratification is optimal for all men and avoid exacerbating existing health disparities [5, 6].

We previously developed a polygenic hazard score (PHS) and demonstrated in an independent European dataset that the PHS was associated with age at diagnosis of clinically significant prostate cancer [1]. Risk stratification with this score also improved the accuracy of PSA testing [7, 8]. We then validated the PHS model (with 46 SNPs) for association with age at diagnosis and with prostate-cancer specific mortality in a dataset that included men of diverse descent, including European, African, and Asian ancestry [2]. We have also improved performance in men of African ancestry by searching for SNPs within that subpopulation [9, 10].

Recent meta-analyses have identified over 200 SNPs associated with prostate cancer, including some identified through subset analyses in men of non-European ancestry [11, 12]. Given the increasing number of SNPs associated with prostate cancer, we evaluated whether including more SNPs in a prostate cancer PHS would improve associations with clinically significant prostate cancer in multi-ancestry datasets.

## Methods

### Participants

Genotype and phenotype data, all de-identified, were obtained from the PRACTICAL consortium. Participants had previously been genotyped via the OncoArray [13] or the iCOGs [14] chips; 90,638 men were available for this analysis.

The available data were split into a training dataset and four testing datasets, taking into account prior power analyses and PHS association results [7, 15, 16]. The training dataset for the model included 72,181 men of European genetic ancestry genotyped via OncoArray (24,010 controls and 48,171 cases). The four testing datasets included: (1) men of African ancestry (*n* = 6253: 3013 controls and 3240 cases), (2) men of Asian ancestry (*n* = 2378: 1184 controls and 1194 cases), (3) the Cohort of Swedish Men (COSM) population-based cohort with long-term outcomes [17] (*n* = 3279: 1116 controls, 2163 cases, and 278 prostate cancer deaths), and (4) the ProtecT population-based prospective trial with screening (prostate-specific antigen, PSA) and biopsy outcomes for both cases and controls (*n* = 6411: 4828 controls and 1583 cases) [18].

### Polygenic hazard score model development using LASSO regularization

We sought to develop an optimal, integrated PHS model with candidate SNPs chosen from those used in the prior PHS and from those identified as susceptibility loci for prostate cancer in a genome-wide trans-ancestry meta-analysis [11]. Candidate SNPs included the 46 from the original PHS development and 269 from the meta-analysis. A machine-learning, LASSO-regularized Cox proportional hazards model approach was used to objectively select SNPs and estimate weights, as described previously [8].

There were 299 unique candidate SNPs associated with prostate cancer consistent across the training and testing datasets used in the present work. We first identified SNP pairs (among the 299 candidates) that were highly correlated (*r*^2^ > 0.95). Each of these paired, correlated SNPs was tested in a univariable Cox proportional hazards model for association with age at prostate cancer diagnosis; the SNP with the larger *p*-value was eliminated from inclusion in the model. All other (unpaired) SNPs were included as candidates for the present PHS model.

The *R* (version 4.0.1) “glmnet” package was used to estimate the LASSO-regularized Cox proportional hazards model [19, 20]. Age at prostate cancer diagnosis was the time to event, and the predictor variables included the genotype allele counts of candidate SNPs and first four European ancestry principal components. Controls were censored at age of last follow-up. The LASSO-regularized model’s hyper-parameter (lambda) was selected using 10-fold cross-validation [19, 20]. The final form of the LASSO model was estimated using the lambda value that minimized the mean cross-validated error.

### Association with prostate cancer

We evaluated the association of the adapted PHS with clinically significant prostate cancer, as well as any prostate cancer, via Cox proportional hazards models in each of the four testing datasets. Clinically significant prostate cancer was defined to be a prostate cancer case with Gleason score ≥7, PSA ≥ 10 ng/mL, T3-T4 stage, nodal metastases, or distant metastases [21].

As the COSM dataset had long-term follow-up data available [17], we additionally evaluated the adapted PHS for association with age at prostate cancer death [16]. There were 278 deaths from prostate cancer in the COSM dataset.

### Hazard ratio performance

Hazard ratios between the top 5% and middle 40% (HR_95/50_), top 20% and middle 40% (HR_80/50_), bottom 20% and middle 40% (HR_20/50_), and top and bottom 20% (HR_80/20_) were estimated for any, for clinically significant, and for fatal prostate cancer. Percentiles of genetic risk were determined within the controls the training set with age less than 70 years [1, 2, 7, 8].

### Family history

Given that family history of prostate cancer is currently one of the most useful clinical risk factors for the development of prostate cancer, we used Cox proportional hazards models to assess family history for association with any, with clinically significant, or with fatal prostate cancer. Family history of prostate cancer was defined as presence or absence of a first-degree relative diagnosed with prostate cancer. Multivariable models using both family history and the adapted PHS were compared to using family history alone via a log-likelihood test with ﻿*α* = 0.01. HRs were calculated for each variable: HRs for PHS in the multivariable models were estimated as the HR_80/20_ in each testing dataset (e.g., men in the highest vs. lowest quintile of genetic risk by PHS). HRs for family history of prostate cancer were estimated as the exponent of the beta from the multivariable Cox regression. As done previously [1, 7], *p*-values were truncated at <10^−16^.

### Positive predictive value performance

Positive predictive value (PPV) performance of PSA testing was calculated using data from the population-based ProtecT screening study [18] (prostate biopsy results were available for both cases and controls with a positive PSA [≥3 ng/mL]). To estimate the PPV and confidence intervals, we generated 1000 bootstrap samples using ProtecT participants with positive PSA, while maintaining the 1:2 case:control ratio in the ProtecT dataset. PPV was calculated as the proportion of PSA-positive participants who were diagnosed with clinically significant prostate cancer on biopsy, looking at those participants in the top 5 (PPV_95_) or top 20 percentiles (PPV_80_) of PHS genetic risk.

### Cumulative incidence curves for PHS

Genetic-risk-stratified cumulative incidence curves for prostate cancer were derived using previously described methods [7, 8]. Briefly, age-specific population data from the United Kingdom (Cancer Research UK [7]) were used to estimate prostate cancer incidence for men aged 40–70 years. Data from the population-based ﻿Cluster Randomized Trial of PSA Testing for Prostate Cancer (CAP) trial [7] were used to adjust this population incidence curve to reflect the age-specific cumulative incidence of clinically significant and non-clinically-significant prostate cancer. Genetic-risk-stratified cumulative incidence curves were then calculated for men in the upper 5 and 20 percentiles of PHS genetic risk by multiplying the prostate-cancer-specific cumulative incidence by the mean value of HR_95/50_ and HR_80/50_ in the testing dataset, respectively.

## Results

A total of 290 SNPs returned non-zero SNP coefficients using the regularization-weight selection and were included in the final model, called PHS290 (Supplementary Material).

HR performance of PHS290 demonstrates risk stratification across percentiles of genetic risk (Table 1). Comparing the top and bottom quintiles of genetic risk for clinically significant prostate cancer, men with high PHS had HRs of 13.73 [12.43–15.16], 7.07 [6.58–7.60], 10.31 [9.58–11.11], and 11.18 [10.34–12.09] in the ProtecT, African, Asian, and COSM datasets, respectively. Similar risk stratification was seen when evaluating risk of any prostate cancer. Finally, when comparing the top and bottom quintiles of genetic risk in the COSM dataset, men with high PHS had a HR of 7.73 [6.45–9.27] for prostate cancer death.

**Table 1:** Hazard ratio (HR) performance in the four testing datasets.

Dataset	Prostate cancer type	HRs and 95% CI			
		HR_20/50_	HR_80/50_	HR_95/50_	HR_80/20_
ProtecT *n* = 6411	Any	0.31 [0.30–0.32]	3.47 [3.36–3.58]	5.93 [5.67–6.21]	11.16 [10.48–11.88]
	Clinically significant	0.28 [0.27–0.30]	3.86 [3.67–4.06]	6.91 [6.42–7.44]	13.73 [12.43–15.16]
African *n* = 6253	Any	0.43 [0.42–0.45]	2.58 [2.50–2.67]	3.69 [3.52–3.86]	5.95 [5.59–6.34]
	Clinically significant	0.40 [0.39–0.41]	2.82 [2.72–2.93]	4.33 [4.10–4.57]	7.07 [6.58–7.60]
Asian *n* = 2378	Any	0.34 [0.33–0.35]	2.99 [2.89–3.08]	5.08 [4.85–5.33]	8.75 [8.21–9.32]
	Clinically significant	0.31 [0.30–0.33]	3.24 [3.12–3.36]	5.66 [5.46–5.98]	10.31 [9.58–11.11]
COSM *n* = 3279	Any	0.33 [0.32–0.34]	3.54 [3.42–3.65]	5.77 [5.51–6.04]	10.87 [10.21–11.57]
	Clinically significant	0.32 [0.31–0.33]	3.59 [3.45–3.75]	5.91 [5.58–6.26]	11.18 [10.34–12.09]
	Fatal	0.38 [0.35–0.42]	2.95 [2.68–3.25]	4.49 [3.93–5.13]	7.73 [6.45–9.27]

HRs are shown with mean sample-weight-corrected values and 95% confidence intervals. Calculations were done using age at diagnosis of any or of clinically significant prostate cancer across all datasets, respectively, and also with age at prostate cancer death for the COSM dataset.

### Family history and PHS290

Family history data based on self-report and were available for 89%, 89%, 43%, and 75% of individuals in the ProtecT, African, Asian, and COSM datasets, respectively; 7%, 18%, 9%, and 14% of individuals, respectively, reported having a first-degree relative diagnosed with prostate cancer. The combination of family history and PHS performed better than family history, alone, for clinically significant prostate cancer (and for any prostate cancer) in each of the four testing datasets (log-likelihood *p* < 10^−16^; Table 2). Additionally, family history and PHS together performed better than family history, alone, for fatal prostate cancer in the COSM dataset (log-likelihood *p* < 10^−16^).

**Table 2:** Multivariable Cox models with both PHS and family history of prostate cancer (defined as ≥1 first-degree relative affected) for association with any prostate cancer, with clinically significant prostate cancer, and with fatal prostate cancer.

Dataset	Variable	Any prostate cancer			
		beta	*z*-score	*p*-value	HR
ProtecT (iCOGs), *n* = 5703	PHS	2.11	72.1	<10^−16^	8.13
	Family history	0.06	1.7	0.1	1.07
African (OncoArray), *n* = 5557	PHS	1.64	53.8	<10^−^^16^	5.03
	Family history	1.13	46.6	<10^−^^16^	3.09
Asian (OncoArray); *n* = 1028	PHS	1.99	68.5	<10^−^^16^	7.64
	Family history	0.45	13.1	<10^−16^	1.56
COSM (OncoArray), *n* = 2453	PHS	2.13	71.2	<10^−^^16^	8.71
	Family history	0.53	19.0	<10^−^^16^	1.70
**Dataset**	**Variable**	** Clinically significant prostate cancer**			
		**beta**	***z*-score**	***p*-value**	**HR**
ProtecT (iCOGs), *n* = 5703	PHS	2.32	49.8	<10^−^^16^	10.01
	Family history	−0.01	−0.2	0.82	0.99
African (OncoArray), *n* = 5557	PHS	1.67	37.4	<10^−^^16^	5.19
	Family history	1.17	33.2	<10^−^^16^	3.22
Asian (OncoArray), *n* = 1028	PHS	1.89	50.4	<10^−^^16^	6.90
	Family history	0.13	2.5	0.012	1.14
COSM (OncoArray), *n* = 2453	PHS	2.13	56.9	<10^−^^16^	6.90
	Family history	0.45	12.6	<10^−^^16^	1.57
**Dataset**	**Variable**	** Fatal prostate cancer**			
		**beta**	***z*-score**	***p*-value**	**HR**
COSM (OncoArray), *n* = 2453	PHS	1.68	19.8	<10^−^^16^	5.51
	Family history	0.43	5.0	4.7 × 10^−^^7^	1.53

Analyses were limited to participants with known family history. Beta and *z*-scores refer to the overall association (within the multivariable Cox regression) with the endpoint of interest, within the corresponding testing dataset. The *p*-values reported are two-tailed from the multivariable Cox models.

### Positive predictive value performance of PHS290

**Fig. 1:**
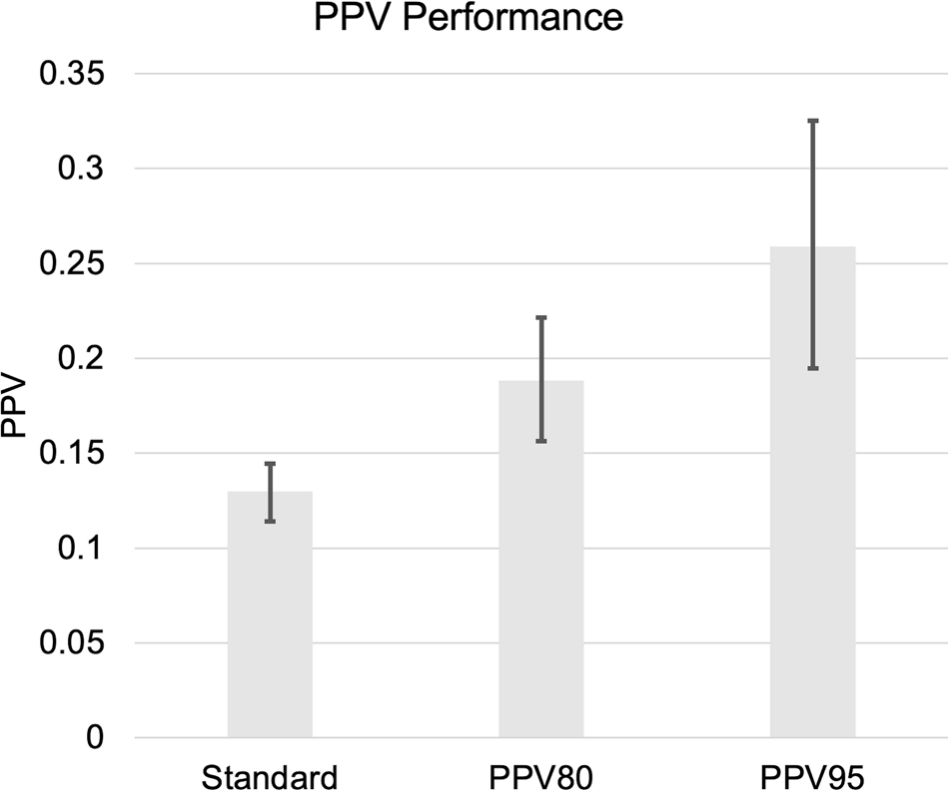
PPV performance in the ProtecT dataset for clinically significant prostate cancer, estimated using 3 approaches: standard (not using PHS), top 20% of PHS values (PPV80), and top 5% of PHS values (PPV95). Error bars are 95% bootstrap confidence intervals.

The PPV of PSA testing for clinically significant prostate cancer was 0.19 (0.15–0.22) for the top 20% of genetic risk (PPV_80_) and 0.26 (0.19–0.33) for the top 5% of genetic risk (PPV_80_; Fig. 1). Both were greater than the overall PPV of PSA alone, which was 0.12 (0.11–0.14).

### Cumulative incidence curves for PHS290

**Fig. 2:**
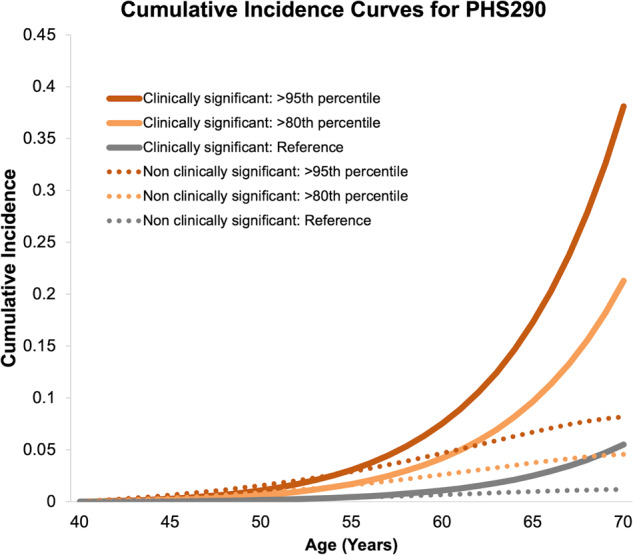
Genetic-risk-adjusted cumulative incidence curves for PHS290. Curves are shown for the upper 5th (>95th) and upper 20th (>80th) percentile of PHS290 for clinically significant and non-clinically-significant prostate cancer. The reference curves represent the overall population average from the UK.

Genetic-risk-stratified cumulative incidence curves for clinically significant and non-clinically significant prostate cancer demonstrate greater prostate cancer incidence with higher genetic-adjusted risk (Fig. 2).

## Discussion

The improved PHS (PHS290) demonstrates excellent genetic risk stratification, including for clinically significant prostate cancer. This was true in four separate testing sets of varied genetic ancestry (Asian, African, European). Additionally, PHS290 was associated with lifetime prostate-cancer-specific mortality in a population-based cohort [16]. Hazard ratios with PHS290 are larger for each of these associations than those reported for previous versions of PHS [2, 8], demonstrating the value of incorporating SNPs from genome-wide meta-analysis and fine-mapping. The improvements demonstrated here are promising for implementing personalized approaches to prostate cancer screening decisions in diverse populations.

Health disparities are a major problem in prostate cancer. Given the exclusion of non-European data in most genome studies [5, 22–24], it is important that the pool of candidate SNPs here included those identified from a recent trans-ancestry meta-analysis [11]. Testing and improving performance of genomic risk scores in diverse populations is critical to equitable implementation of these new tools and important for avoiding exacerbation of existing disparities. PHS290 still performs better in men of European genetic ancestry—an expected result, given the much greater data availability in that population. Further genomic studies in diverse populations are essential, as diversity in model development improves performance in diverse populations [9, 10].

The intersection of social constructs like race/ethnicity and genomics also raises interesting and entangled challenges. Even availability of genomic data is only part of the problem, as disparities in health outcomes are rooted in systemic racism and inequities in access to healthcare [25, 26]. Genotypic ancestry may be a step toward biology, but the continental groups still represent an oversimplification of genetic diversity and a pre-determined assumption that socially defined categories have biological meaning in all contexts. We have previously shown that agnostic genetic clusters are informative for subgroup analyses [2], and this approach may be a better way forward, provided the genomic diversity of the whole population is represented in the available data. Here, we have used genotypic ancestry to evaluate the potential differential performance in groups historically excluded from large-scale genomic studies. We also note that local ancestry may be a critical consideration in admixed populations. We have found previously that PHS performance can vary by the makeup of a *region* of the genome, beyond what is explained by *global* ancestry categories assigned for an individual’s entire genome [10]. Despite these challenges and opportunities for future improvement, the current results demonstrate PHS290 does provide meaningful risk stratification in diverse datasets.

The cumulative incidence of clinically significant prostate cancer is heavily influenced by age-specific genetic risk, as demonstrated by genetic-risk-stratified cumulative incidence curves (Fig. 2). As men with high PHS290 age, the incidence curve for clinically significant prostate cancer increases dramatically, prominently separating from the incidence for non-clinically-significant prostate cancer. This effect is driven by the high HR for clinically significant prostate cancer in these men, combined with increasing incidence specifically of more clinically significant cancers as men age [7, 27]. Furthermore, we found that risk stratification with PHS290 improved accuracy of PSA testing, as assessed by probability of a positive PSA test leading to a diagnosis of clinically significant cancer on biopsy. Consistent with a prior study [8], this improvement in PPV of PSA testing was not better when using PHS290 than when using PHS46 in this dataset [2]. PPV analyses in larger datasets could permit finer granularity for age-specific genetic risk to assess whether the increased HRs of PHS290 might translate to better performance of PSA testing than that achieved already with PHS46.

The HRs reported here suggest clinical relevance for PHS290. Predictive tools in routine clinical use for other diseases (e.g., breast cancer, diabetes, and cardiovascular disease) have reported HRs of ~1–3 for endpoints of interest [28–31]. Current guidelines recommend earlier and more frequent consideration of prostate cancer screening for men with a family history of prostate cancer or African ancestry, citing an elevated risk 28–80% above that of men without these risk factors [32–34]. Guidelines more strongly recommend earlier and more frequent screening in men with germline mutations in *BRCA2*, which are rare but are estimated to infer up to 3-fold increased risk [33–35]. In the present study, men in the top 20% for PHS290 (compared to men with *average* risk) had HRs for clinically significant prostate cancer of ~2.8–3.9. For men in the top 5% for PHS290, those HRs increase to 4.3–6.9, depending on ancestry. While individuals with high polygenic risk may also develop low-grade prostate cancer in their lifetime, the time-to-event analysis applied here shows that high genetic risk confers a greater hazard for prostate cancer death. This finding is consistent with prior reports, though the effect size is larger with PHS290 [2, 16, 33].

Family history data were not uniformly available across source studies or testing datasets and were notably less available in the Asian dataset (43%, compared to ≥75% in the other testing datasets). The proportion of individuals with positive family history—among those with available family history data—also varied across datasets, ranging from 7% (ProtecT) to 18% (African). The interplay of family history and polygenic risk warrants further investigation in more complete cohorts. It is also worth noting that family history availability is not always available (or reliable) in clinical practice. Nonetheless, both family history and polygenic risk appear important for assessing individual risk of prostate cancer. Possibly, family history represents not only inherited genetic risk but also shared environmental exposures.

If this technology is to be implemented in clinical practice, it is important to consider reliability. Genotype arrays are known to have excellent reproducibility, with concordance of 99.40–99.87% on repeat testing with the same array [36]. Concordance across genotyping platforms is also excellent at 98.80% [36].

Our work has some limitations. First, the weights were calculated in men of European genetic ancestry alone, although SNP candidate selection was performed in multi-ancestry analyses. Future studies evaluating PHS for prostate cancer risk stratification will include non-Europeans in SNP weight calculations. The available data did not permit testing of PPV or association with fatal disease in non-European populations. Moreover, the cumulative incidence curves here are specific to the UK, where we had the most robust population age-specific incidence data for clinically significant prostate cancer. The testing sets used in the present study did represent a very small proportion of the data used for candidate SNP identification in the prior genome-wide association meta-analysis (as opposed to the development of PHS46, which was performed in a dataset completely independent of the validation dataset) [2, 11]. However, the training and testing sets were kept separate in the present study, and the use of the LASSO-regularized Cox model reduces over-fitting [37].

The PHS290 described here has the strongest reported association with prostate cancer in men of European, African, and Asian genetic ancestry. The score was also associated with lifetime prostate-cancer-specific mortality in a population-based cohort. A performance gap remains between genetic ancestry groups that might be closed through development using more data from men of Asian or African ancestry. Nonetheless, the results here suggest PHS290 may improve prostate cancer risk-stratification efforts in multi-ancestry populations.

## Supplementary information

Supplementary Material

## Data Availability

Prostate Cancer Association Group to Investigate Cancer Associated Alterations in the Genome (PRACTICAL) Consortium data are available upon request to the Data Access Committee (http://practical.icr.ac.uk/blog). Questions and requests for further information may be directed to PRACTICAL@icr.ac.uk. Code used for this work is stored in an institutional repository and will be shared upon request to the corresponding author.
